# It is time to combine the two main traditions in the research on the neural correlates of consciousness: C = L × D

**DOI:** 10.3389/fpsyg.2014.00940

**Published:** 2014-08-22

**Authors:** Talis Bachmann, Anthony G. Hudetz

**Affiliations:** ^1^University of TartuTallinn, Estonia; ^2^Medical College of WisconsinMilwaukee, WI, USA

**Keywords:** consciousness, neural correlates of consciousness, contents, level, state, visiual perception, thalamus, excitatory postsynaptic potentials

## Abstract

Research on neural correlates of consciousness has been conducted and carried out mostly from within two relatively autonomous paradigmatic traditions – studying the specific contents of conscious experience and their brain-process correlates and studying the level of consciousness. In the present paper we offer a theoretical integration suggesting that an emphasis has to be put on understanding the mechanisms of consciousness (and not a mere correlates) and in doing this, the two paradigmatic traditions must be combined. We argue that consciousness emerges as a result of interaction of brain mechanisms specialized for representing the specific contents of perception/cognition – the data – and mechanisms specialized for regulating the level of activity of whatever data the content-carrying specific mechanisms happen to represent. Each of these mechanisms are necessary because without the contents there is no conscious experience and without the required level of activity the processed contents remain unconscious. Together the two mechanisms, when activated up to a necessary degree each, provide conditions sufficient for conscious experience to emerge. This proposal is related to pertinent experimental evidence.

## INTRODUCTION

Scientific research on consciousness has made a major leap forward after influential papers by Bernard Baars, Francis Crick, and Christof Koch outlining the logic and perspective directions of the contrastive analysis (Baars, 1989; Crick and Koch, 1990, 2003). To discover the neural correlates of consciousness (NCC), they suggested to compare brain-process recordings collected in the conditions when subject is conscious (C) with those when that subject is not conscious (U). The difference between these two conditions was operationalized as the NCC, the minimal necessary neural correlates expressed by specific signatures of brain processes differentiating these two conditions as NCC = C - U. There are two main traditions of research using this methodological approach: (1) studying the general states of consciousness versus unconsciousness for revealing NCC (Tradition-1) and (2) studying the correlates of the contents of consciousness in a conscious subject who in some of the experimental trials (or subconditions of trials) has subjective experience of the target stimulus and in some other conditions does not (Tradition-2). (See articles by Steriade, 1996; Laureys, 2005; Massimini et al., 2005; Seth et al., 2006; Rosanova et al., 2012; Sarasso et al., 2014 for an overview of typical tradition-1 studies and Del Cul et al., 2007; Aru and Bachmann, 2009a,b; Lamy et al., 2009; Hesselmann et al., 2011; Pitts and Britz, 2011; Aru et al., 2012a; Sekar et al., 2013 for typical tradition-2 studies.) Tradition (1) has made some progress in finding NCC for the *state* of consciousness while tradition (2) has found a lot of data on NCC in terms of its specific *content*. Ironically, an observer cannot infer the state of consciousness with certainty without testing the presence of some subjective content in consciousness. In other words, there is no empty or contentless consciousness and even though a highly skillful meditation may perhaps reduce the contents to a minimal extreme (Hohwy, 2009), an experience of some unchanging “empty” state of mind will still have a felt emptiness or oneness as a content. Thus, states and contents seem to be unseparable in that as soon as the unconscious state changes to a conscious state, some content of consciousness inevitably appears. Nevertheless, the above mentioned two traditions of research have been developing in relative mutual isolation.

Recently, a methodological crisis in studying the NCC was spotted (Aru et al., 2012b; de Graaf et al., 2012). It appears that with standard contrastive analysis not only the NCC of the consciousness itself are extracted from brain recordings, but the observed neural signatures may also pertain to processes necessarily preceding those directly responsible for consciousness itself (correlates of the prerequisites of consciousness or NCCpr) and processes that are aftereffects or consequences of consciousness (NCCae). It appears that it is not at all easy to distinguish the NCCpr and NCCae from the processes directly equivalent to the neural processes minimally sufficient for the conscious experience itself (i.e., the NCC proper, or constituents of consciousness, to use the distinction made by Miller, 2001, 2007). We believe – together with Hohwy, 2009 – that the theoretical picture used to explain and understand the results of research on NCC is confused partly because the above mentioned two traditions, the study of conscious state vs. content, have progressed separately. “It seems then that neither the content nor the state-based approach taken in isolation from one another will help us discover the NCC” (Hohwy, 2009, p. 435). Thus, we argue that Tradition-1, by concentrating on the brain mechanisms necessary for the general state of consciousness, has been neglectful with regard to the systematic experimentation in the domain of finding the NCC for subjective contents and because of this, it has had difficulties in properly specifying the NCC. Neither the difference in the exact contents nor the difference in the exact timing of the subjective experience of these contents along the subsecond timescale have been studied. We also argue that Tradition-2, by concentrating on the brain processes that correlate with subjective awareness of specific target stimuli has tended to overlook the data and regularities obtained in the research on NCC of conscious states. In observing and analyzing brain-process signatures as candidates for NCC, no attempt has been made to clearly distinguish the contributions of the state and content systems to these signatures.

Aside from the issue of the exact timing of emergence of conscious contents and their NCC in terms of brain-imaging signatures, an attempt to bridge to state-content interrelationship is the former study by Owen, Laureys and colleagues capitalizing on the contrastive analysis of fMRI signals between vegetative state (VS) patients and normal alert controls (Owen et al., 2006). VS patients who were clinically defined as being unconscious were asked to imagine playing tennis while their pattern of fMRI activation was compared to that obtained in healthy control subjects reporting the corresponding phenomenal content. The two brain activation patterns were quite similar, which the authors used to suggest that VS patients have conscious contents. Hohwy (2009) criticizes this approach based on the grounds that what the investigators tested really was volition rather than qualia. In our opinion this critique is insufficient for several reasons: (1) the subjective experience of volition is also a kind of consciousness with its content, although possibly different in form from sensory experience fo qualia, (2) in order to express volition, patients had to first semantically process the command and be able to differentiate between the requested choices. Thus it seems that either we really have some residual form of consciousness in VS patients or the brains of VS patients are capable of surprisingly intricate and precise information processing without the concomitant subjective experience simply executing automatisms of processing sensory input up to cognitive and semantic stages (supporting the zombie mode of complex information processing). On the other hand, the so-called zombie modules seem to exist indeed if we accept data by Kotchubey (2005) and Owen et al. (2006) as evidence for complex perceptual-cognitive processing of stimulation contents by the unconscious brains. The serious shortcoming of this type of research consists in the lack of reliable independent measures clearly associated with phenomenal subjectivity. We do not know whether the specific contents processed by the brain in a specific state (e.g., VS or a locked-in state) are also reflected phenomenally in the first person perspective. Because verbal reports from subjects in states for which we cannot definitely say whether these are conscious or unconscious cannot be reliably obtained, we are left with the option to study *the effect of experimental manipulations of the brain’s state systems only when these manipulations are applied when the human subject is in a state allowing subjective report*. When this type of research has developed well enough and the corresponding mechanisms of consciousness will have become more precisely specified, experiments with animal models can be then carried out in a more meaningful way with regard to the problem of NCC.

Although many of the clinical criteria for qualifying a state as a conscious, minimally conscious or unconscious state and as a state of sleep or behavioral arousal are objectively physiological (e.g., EEG) and behavioral (e.g., reflexes), at present we can rely on subjective reports and evaluations of the contents of mind as the only available method to assess presence of consciousness reliably. An important dimension which allows measurement of variable states from the point of view of subjectivity is the scale of the *levels of consciousness* expressed in terms of the clarity, vividness and fullness of subjective conscious experiences. Vague, barely noticeable, fragmented, poorly differentiated experiences stand at the one end of the scale and clear, vivid, differentiated and stable experiences at the other end. Sedated patients typically describe their experiences in terms of this kind of qualia continuum where the quality of contents changes along with the change of state from unconsciousness to hypnagogic experiences to vague experiences up to the full blown clear and vivid perception of the world and private thoughts (Newton et al., 1990; Andrade et al., 1994; Zacny et al., 1994; Hudetz and Pearce, 2010). Consciousness contents have different levels of expression that can vary vith invariant environmental stimuli input as a function of the varying state of the brain. Thus, operationally the effects of the state system on the content system should be possible to measure as the variations in the level of subjective experience as measured by the contents typical for the gradations of the level (Bachmann, 2012). Importantly, there are scalable attributes of the level common to different modalities and within-modality contents which makes these attributes universal.

We believe that by combining the two approaches within a theoretical synthesis some – but not all – of the controversies in NCC research can be solved. We suggest a theoretical synthesis where the activity of the mechanisms examined in each of the two traditions are the prerequisites (NCCpr) of consciousness, but together they form the combination jointly sufficient for consciousness (i.e., NCC proper). In a highly simplified form, we propose an expression for consciousness: C = L × D, where C stands for consciousness, L stands for the critical *level* of activity of neuromodulatory systems regulating the state of the brain, and D stands for the *data*, i.e., the contents of consciousness provided by the specific representational systems of the brain. In order to ease the abstractness of the concept, we give a few examples on what we mean here. In an experiment aimed to manipulate the state level L, for example, with an incremental dosing of anesthetics, a suitable task-response can be studied simultaneously at matched performance levels (accompanied with subject’s scaling of the level and/or contents of his/her subjective experience) while doing fMRI, MEG, EEG or something else. In doing this, matching the task performance may be important to minimize the contribution of NCCae on the recorded neuronal signatures. One could also look at other changes in the brain, e.g., functional connectivity as candidates of NCC proper or its reliable signature. Similarly, EEG/ERP recordings can be used for comparing subjective reports under varying degrees and means of sedation by anesthetics, including measurements of possible NCCae (e.g., Veselis et al., 2009). Some additional examples will be given later on in this article. Obviously, in order to follow the strategy of the combined use of the L-system and D-system based approach, these systems should be known sufficiently well in terms of neuroanatomy and ways of functioning.

Before elaborating this concept further, let us have a closer look at the putative brain mechanisms and processes that have been proposed as necessary for consciousness both in terms of its content and state, and after having done so, we will return to the problem of NCC.

## TWO MUTUALLY INTERACTING BRAIN SYSTEMS RELATED TO CONSCIOUSNESS

To experience something subjectively, the brain has to be in the state of consciousness, but also capable of processing sensory data which constitute the contents of experience. In other words, the presence of specific sensory contents in conscious awareness is not automatically granted, but requires involvement of cerebral *interactive mechanisms* that aid the explication of preconsciously processed information. Neurobiological experimental data, deep brain stimulation data, and anesthesiological data converge in showing that modulations mediated by the thalamocortical “nonspecific” system targeted at the specific system of encoding the sensory-perceptual information are necessary for subjective contentful experience to emerge (Brooks and Jung, 1973; Bachmann, 1994; Purpura and Schiff, 1997; Llinás and Ribary, 2001; Ribary, 2005; John, 2005; Hudetz, 2006; Alkire et al., 2008; Hudetz and Alkire, 2011; Giacino et al., 2012; Liu et al., 2013). An important aspect of this dual functional architecture consists in the way these two systems interact at the cellular level. There is massive presynaptic targeting of the apical dendrites of the layer-5 pyramidal neurons in cortex originating from the non-specific system while the layer-5 pyramidal neurons are the prime part of the specific afferent system at the cortical level serving the function of encoding specific stimulation content. This specific afference arrives presynaptically at the more somatic parts of these nerve cells. It is suggested that this is the cellular level mechanism where nonspecific and contextual top-down modulatory influences interact with primary specific afferent input and that the extent or pattern of this interaction highly correlates with subjective states of consciousness (Brooks and Jung, 1973; Purpura and Schiff, 1997; Llinás and Ribary, 2001; Ribary, 2005; Alkire et al., 2008; Pillay et al., 2014). The layer-5 pyramidal neurons are naturally suitable to be a mechanism where perceptual contents are modulated up to consciousness, but at the same time this mechanism allows both pre-conscious and unconscious information processing. Although inputs to cell soma and dendrite tuft can perform their own functions autonomously (Berger et al., 2001; Larkum et al., 2004), these inputs are also capable of intense integrative interaction. Therefore, the same system can perform information processing in either the preconsious (non-interacting) or the conscious (interacting) domain. Membrane activity caused by somatically targeted sensory input is “available” for effective (i.e., hypothetically awareness-providing) modulation if it has sufficient frequency (Larkum et al., 1999a) and optimal timing with respect to the apical dendrite targeted input (Larkum et al., 1999b; Larkum, 2013). The autonomous function of the two neuron compartments is possible also due to the fact that the level of membrane depolarization is progressively attenuated distally from soma, and also from the tuft, as well as for the very fact that an absence of temporal coincidence of somatic and distal-dendritic input dissociates the two subcellular activities and thus the potential to act as an integrative device remains unused (Berger et al., 2001; Larkum, 2013).

For conscious perception, it is *necessary that both systems* be active in concert – (i) the bottom-up, specific content-signaling data system D receiving afferents by the layer-5 pyramidal neurons’ basal compartment, and (ii) the modulation system sending intracortical top-down associative signals and/or non-specific thalamo-cortical signals leading to up-states necessary for a sufficient consciousness level L (Bachmann, 1994; Ribary, 2005; Larkum, 2013). When only one of the two principal input streams to pyramidal neurons – somatic specific afferents and dendritic presynaptic nonspecific afferents – is active without the “partnering” effect of the other system, consciousness is expected to fade away. Formally: D(0)****× L(z) = C(0); D(x) × L(0) = C(0). Only D(x) × L(z) = C(x), with x being the contents of the experienced subjective state.

It is likely that for the hypothetical dual-input consciousness mechanism to become ignited, both neuron compartments – somatic and apical dendritic – must receive enough presynaptic input in order to generate the wide excitatory postsynaptic potential (EPSP) plateau-waves carrying a burst of spikes representing an up-state. One good candidate for being the core cellular level mechanism in this system is the backpropagation activated calcium spike firing (BAC firing) mechanism described by Matthew Larkum and colleagues. For the layer-5 pyramidal neurons to generate plateau-wave-based spiking, the temporal coincidence of somatic sodium channel-related presynaptic input (i.e., data for the subsystem D) and calcium channel-related presynaptic input targeted at the apical compartment of the cell dendrite (i.e., modulation by the subsystem L) is necessary (Larkum et al., 1999b; Larkum, 2013). It is important to acknowledge that dendritic calcium spikes are a direct target of anesthetics (Potez and Larkum, 2008) and after release from the anesthetic effects, calcium electrogenesis in layer-5 pyramidal neurons dramatically increases (Murayama and Larkum, 2009). This supports the notion of the principal importance of the BAC firing mechanism for consciousness. Importantly, suprathreshold input to the neuron’s body (responsible for the bottom-up inflow of sensory signals by D) produces fewer action potentials of the cell than triggering of the dendritic Ca^2+^ spikes. This once more substantiates the importance of modulatory brain processes in addition to the straightforward sensory afference and provides a convincing argument for the common effects of biased perception being under the contextual and arousal systems control. [See also works by E. Roy John (e.g., John, 2005) and Rodolfo Llinás (e.g., Llinás et al., 1998) on the putative significance of coincidence detection for the effectively working consciousness mechanism.]

In addition (or alternatively to) the thalamocortical resonance or interaction theory, the role of cortico-cortical interactions in the NCC should not be excluded or overlooked. For example, Mashour and coworkers (Lee et al., 2013) demonstrated the selective alterations in fronto-parietal interactions during general anesthesia with three different anesthetic agents. The use of different drugs in the same investigational setting is important in order to be able to identify an anesthetic-invariant mechanism of loss and return of state consciousness (John, 2001), removing agent-specific effects, and by the same token, at least some of the NCCpr or NCCae. A particularly interesting aspect of the work of Lee et al. (2013) is that fronto-parietal communication was preferentially suppressed by all three anesthetics in the feedback (top-down) direction. This important finding is consistent with what was proposed by Del Cul et al. (2007) and predicted by prior preclinical anesthesia studies by Imas et al. (2005a), suggesting that cortical long range connectivity, especially in its top-down aspect, may be critical for consciousness. On the other hand, based on the general knowledge that non-specific thalamocortical effects target more frontal and central than posterior areas it is possible that the typical effect on the top-down aspect of cortical effective connectivity reflects also (or first of all) the effect of the extended thalamo-cortical modulation system. Further, indirect support for the nonspecific brain systems regulating conscious thresholds for visual stimuli comes from a recent study by Park et al. (2014) who showed that the threshold varied with the phase of heartbeats. It is difficult to assume that heartbeats are directly associated with processing specific visual input, but it is easy to understand that and how heartbeats reflect activation levels of the non-specific systems involving the autonomic system contribution to arousal.

In some situations, specific input is processed by the brain and/or stored in its long term memory system but there is insufficient L-system modulation for conscious awareness of the sensory input or stored memory-information. Examples include subjects having fallen asleep, undergone fainting, or brought into anesthetic states. Brains of sleeping or anesthetized subjects receive localized specific input that comes from sensory receptors, but associative input and modulating arousal input from the reticulo-thalamic activation system is insufficient (Magoun, 1958; Hassler, 1978; Purpura and Schiff, 1997; John, 2005; Ribary, 2005; Hudetz et al., 2009; Hudetz and Alkire, 2011; Zhou et al., 2011). Information integration to consciousness is absent (Alkire et al., 2008). On the other hand, when the L-system is artificially stimulated, arousal and consciousness accompanied by sensing the contents of ambient stimulation can be brought about, cortical information-integration can be augmented, release from visual masking produced, or artificial sensations evoked (Smirnov et al., 1978; Tasker et al., 1980; Bachmann, 1994; Giacino et al., 2012; Pillay et al., 2014). Artificial stimulation of the intralaminar thalamic sites that are part of the nonspecific system augments visual evoked potentials in response to grating stimuli (Hunsperger and Roman, 1976). Visual, auditory and somatosensory research using recording of brain potentials has repeatedly shown that the level of expression and timing of the negative-polarity potential N1 (with post-stimulus latency equal to about 50–200 ms depending on the modality and stimulation characteristics) strongly correlates with conscious perception (Uttal and Cook, 1964; Wagman and Battersby, 1964; Hassler, 1979; Alter et al., 1990; Cauller and Kulics, 1991; Bachmann, 1994; Imas et al., 2006; Schubert et al., 2006; Schoenfeld et al., 2011; Auksztulewicz et al., 2012; Auksztulewicz and Blankenburg, 2013; Sinke et al., 2014; Pitts et al., submitted). For example, while early post-stimulus potential components are more or less invariant between sleep or sedation on the one hand and awake conditions on the other hand (or even increased in sedation – Imas et al., 2006), N1 is considerably suppressed in the unconscious state. Similarly, visual cortical neurons in anesthetized animals show early activity in response to light flashes similar to the unanesthtized state, but long-latency responses significantly decrease under sedation (Hudetz et al., 2009). Also, in the crowding effect when target information is left out of awareness, N1 is suppressed although P1 ERP component still reflects basic stimulus characteristics regardless of crowding (Chicherov et al., 2014). When similar monocular textures fuse into subjectively visible images, conspicuous occipitally recorded negative component is present, but absent when fusion into visibility is not achieved (Fahrenfort et al., 2012). Moreover, recently it was shown that whether subjective experience of a visual stimulus in hysterical blindness is present or not is reflected in the amplitude of N1 (Schoenfeld et al., 2011). Importantly, while N1 appeared as an authentic NCC, fMRI recording data could not differentiate between consciously seen vs unseen stimuli. For our purposes it is essential that the electrogenesis of negativity of the surface-recorded brain potentials considerably owes to the apical dendritic activity in the upper layers of the cortex (Larkum, 2013). The stimulus-evoked negativity can be easily explained as resulting from the excitatory synaptic input to the distal compartment of the apical dendrites of layer-5 pyramidal neurons. The fact that consciousness of a stimulus emerges with a relatively long delay after 100 ms (Bachmann, 1994, 2000) is strongly consistent with the fact that N1 is relatively slow compared to the timing of the early arrival of stimulus signals to cortex as indexed by the faster ERP components. This is despite the fact that the early ERP components with latencies under 100 ms can vary depending on whether the target stimulus presented in invariant physical conditions becomes consciously experienced or not (Aru and Bachmann, 2009b) suggesting that they are signatures of the NCCpr and not NCC proper. Furthermore, an ERP signature termed visual awareness negativity (VAN) most conspicuously present as recorded by lateral occipital electrodes also correlates with stimulus awareness (Railo et al., 2011). (However, see Bachmann, 2009, concerning the problem that VAN duration is too short compared to the duration of subjective experience when subjects hold target-stimulus in explicit immediate memory. This problem appears to find a solution in the correlation of slow negative potentials with conscious awareness as discussed later in this article.)

Thus, taking into account the experimental regularities reviewed above, let us operationally regard EEG potential negativity as a signature of the involvement of the L-system in modulating the neural activity of the D-system. This notion does not mean that modulation by the L-system *always* leads to conscious experience of the stimuli; it is likely that a certain level of the L-activity modulating D-activity is necessary. This in turn implies the existence of a threshold level and/or threshold duration of the processes producing ERP negativity, with sub-threshold levels of the measured negative cortical potential measurable in principle. Consistent with this, Intaité et al. (2014), demonstrated pre-reversal negativity as a signature of the change in conscious perceptual contents. Several hundred ms before subjective reversal of the perceptual contents of an invariant ambiguous stimulus, ERP negativity was enhanced when subsequently the conscious contents were changed.

Not only brief transient ERPs with negative polarity correlate with stimulus awareness timing, but also the slower negative deflections of the brain potentials such as, the contingent negative variation, slow cortical potential waves, or contralateral delay activity appear to be good candidates of a reliable and robust correlate of consciousness, especially as related to expectancy states and application of general-purpose brain-process resources for a specific task involving focused attention (Rösler et al., 1997; Birbaumer, 1999; Devrim et al., 1999; He and Raichle, 2009; Murayama and Larkum, 2009; Fischer et al., 2010; Murd et al., 2010; Stamm et al., 2011; Birbaumer et al., 2012; Pun et al., 2012; Jo et al., 2014; Li et al., 2014). Basically, the slow potential dynamics reflects changes in cortical excitability (He and Raichle, 2009; Raichle, 2011). This notion is substantiated by the known intimate relationship between the slow cortical potential and the thalamo-cortical neuromodulatory system (i.e., the system controlling L, according to our notation). This conceptualization is consistent with observations that trans-cranial magnetic stimulation (TMS) of the occipital cortex elicits state-dependent effects when performed in a state of increased arousal (brought about by caffeine administration) as compared to quiet wakefulness (placebo condition; Murd et al., 2010), or during NREM sleep compared to awake state (Stamm et al., 2011). When caffeine was applied or wakeful state was tested, TMS evoked a stronger early negative component (N1) and enhanced slow negativity in remote cortical areas compared to the placebo condition or NREM sleep condition. Because TMS is a task-irrelevant, nonspecific signal in terms of the contents of consciousness, these results speak in favor of the stance that (slow) negativity reflects nonspecific effects on cortical excitability. Provided that slow negativity correlates with (readiness for) long range effective connectivity, TMS is expected to facilitate cortical communication in conscious states in order to allow sufficient information integration. Studies by Tononi, Massimini and associates indeed show this (e.g., Massimini et al., 2009; Ferrarelli et al., 2010). They tested the state-dependence of the cortical propagation of a TMS-induced EEG signal and found that in the unconscious condition, either in slow wave sleep or anesthesia, long-range cortical communication was suppressed, and restricted to a local region surrounding the stimulation site. In terms of temporal dynamics, the late (sustained) component of TMS-evoked signal was also suppressed, in agreement with results obtained previously with the direct measurement of flash-evoked unit activity in the visual cortex of rats (Hudetz et al., 2009).

In accordance with the conceptualization presented above, Yasuda et al. (2011) showed that with the loss of consciousness at sleep onset, N1 decreased, P2 increased (!), and CNV was increasingly more positive. In sleep the CNV was absent.

We believe that a step forward in revealing the NCC would be achieved by the use of a combined strategy consisting in three key requirements: (i) *causal* mechanistic effects are used and studied instead of correlational ones in the objective domain of research so that microelectrode stimulation, optical stimulation, TMS, psychopharmacological intervention, or other methods of purposeful manipulation of neural tissue in selected brain areas allow to study the resulting effects on brain processes by brain-imaging or similar methods; (ii) the *relative involvement of the D- and L-systems* is specifically examined in the context of objective causal manipulations and measurements; (iii) psychophysical procedures are used in combination with (i) and (ii) allowing the *precise measurement of subjective contents* of *consciousness* as they emerge, unfold, and decay along the objective time axis. Correspondences between precise timing of the neural signatures and psychophysical phenomena pertaining to the domains of (i), (ii), and (iii) will hopefully make it possible to find causal relationships that are more likely to be indicative of the real constituents of consciousness in terms of the underlying neural processes. An important accompanying research agenda should be to use animal models with mammalian species, executing studies (i) and (ii) and by analogy, invoking and postulating the corresponding variability of the experienced contents (iii). In what follows, we will provide a few *ad hoc* interpretations of the published research suitable for differentiating the D- and L-system effects in the context of the problem of NCC. Thereafter, we will conclude by envisaging possible experimental approaches for future research in service of the view that C = L × D.

## EMPIRICAL FACTS CONSISTENT WITH C = L × D

There are surprisingly few research data on the NCC as based on experiments explicitly relating content- and state-related system effects within the same study that utlize the methodology of contrastive analysis. Measurements and discussions have been evolving around the specific D-system contributions with rarely – if at all – mentioning the L-system contribution. This conclusion is especially valid with regard to the real-time examination of the NCC as they unfold along the fine timescales of psychophysical experimental paradigms. Although a lot of psychophysical and cognitive neuroscience research has been published with results that can be *interpreted* according to our C = L × D “formula,” this kind of research lacks the power of *direct evidence*. Nevertheless, here we discuss both – studies with experimental protocols and designs more directly combining the L-system and D-system variables as well as studies allowing indirect interpretations of their data in terms of L- and D-system interactive contributions. We do this, being well aware of the methodological weaknesses of the *ad hoc* type of argumentation about the second sub-variety of the pertinent research as mentioned above.

Alert patients regularly treated by chronically implanted microelectrodes in order to relieve the suffering from Parkinson’s disease were used as volunteer subjects in a visual mutual masking experiment (Bachmann, 1994). They were asked to discriminate spatially overlapping brief stimuli presented successively with stimulus onset asynchrony (SOA, values less than 100 ms) leading to masking. Compared to the control condition without the pre-test microelectrode stimulation, awareness of the visual targets improved considerably in the main condition with a preliminary activating stimulation targeted at the thalamic nuclei which were the known part of the L-system (including the central medial intralaminar nucleus). Artificially activating the L-system produced improvement of perception of the specific contents represented by the D-system. The effect was obtained also with invariant physical conditions of the independent variables.

A very brief near-threshold Gabor patch was presented for orientation discrimination in the study by Li et al. (2014). MEG responses as an equivalent of slow cortical potential (SCP) were recorded in the conditions allowing comparison of trials with (i) strong and marginal awareness and unawareness, (ii) correct and incorrect behavioral responses, and (iii) subjective confidence in the correctness of response, all with invariant values of the principal independent variables. After the effects of objective performance and confidence were both removed from data, the SCPs were found to be a strong and reliable correlate of consciousness while the neural signatures correlating with veridicality of response and confidence were mostly related to transient brain activity. Because long-lasting EPSPs at the apical dendrites of the (mostly layer-5) pyramidal neurons are considered as the main contributors of surface-recorded SCPs and as the equivalent MEG contrasts between aware/unaware conditions stood for the expression of SCPs, we can conclude that modulation of the D-system (carrying the contents of Gabors) by the L-system (modulating the EPSPs of the content-carrying neurons of the D-system) may be an instance of the NCC. In light of these results, it is inviting to speculate about the possible reasons why LFP responses indicative of correct stimulus perception in the slower theta band preceded the single-neuron gamma band response by about 50-100 ms in the experiment reported by Rey et al. (2014). It is possible that the slower LFP activity in the theta band range represents the L-system activity and the gamma band single-cell responses correspond to the D-system activity. If so, the contextual and/or expectancy-related or arousal-related L-system activity may precede the bottom-up afferent signaling from the specific modal stimulus so that it enhances EPSPs by presynaptic signals targeting the apical dendrites, but not much the high-frequency firing which needs also a sufficient input to the somatic compartment of the D-system nerve cells in the temporal cortex. Only as soon as there is enough coincidence of the L-system and D-system input, firing commences and explicit recognition results. This conjecture is consistent with that of Larkum (2013) with respect to the BAC firing mechanism.

Thus, in spite of suggestions by several authors (Llinás, for example) it seems that a change in thalamocortical 40Hz gamma oscillations, at least in the local sense, may not be the critical factor in the involvement of the L-system for the NCC. Various data support this. In anesthesia with various select agents, the power of both spontaneous and sensory evoked gamma oscillations is *enhanced* (Imas et al., 2005b; Sellers et al., 2013), although preliminary findings suggest that high-frequency gamma (70-140 Hz) may be suppressed (Hudetz et al., 2011). Nevertheless, the long-range cortical interactions at 40–50 Hz gamma frequencies do seem to be selectively altered in an opposite direction to what should have been expected from the traditional gamma band hypothesis (Imas et al., 2005a,b). All this is consistent with recent findings by Aru et al. (2012a) and Hermes et al. (2014) with respect to the lack of a band-specific gamma correlate of conscious perception. As suggested by several authors (e.g., He and Raichle, 2009; He, 2014; Miller et al., 2014), broadband and/or scale-fee activity of neuronal populations seems to be associated with surface potentials, dendritic integration and very likely with awareness. It appears that BAC firing as a potential mechanism subserving the NCC are unlikely to be tightly coupled with the gamma band responses in implementing the C = L × D type of interaction.

The BAC firing mechanism as related to behavior of alert animals was involved also in the findings of Palmer et al. (2012a,b). They showed how contralateral sensory stimulation inhibited the firing of layer-5 pyramidal neurons in response to a sensory stimulus *in vivo* and that this inhibition acted on the apical dendrites. Somatic input to pyramidal neurons generates predominantly short-lived EPSPs and fragmented cortical neural activity but not a sustained “field of consciousness,” unless substantial additional modulatory input to the apical parts of the dendrites arrives. On the other hand, only or predominantly non-specific modulation by the L-system targeting the superficial cortical layers in the absence of sufficient afferentation from sensory signals by the D-system (targeting the perisomatic compartment) should lead to fading or “contentless” conscious experience. For example, this happens with fading of retinally stabilized images (due to massive adaptation of the same sensory units), Ganzfeld effects featuring feelings of sensory emptiness (e.g., the coloring of the Ganzfeld disappears from subjective phenomenal experience), falling asleep in sensory isolation experiments, and with hypnotic effects of mononotonous and predictable stimuli (the responses to which are diminished by adaptation effects).

Whatever the normally functioning consciousness mechanism is, it must not represent mainly unreal or hallucinatory content in the awake state but perform adaptively sound *reality monitoring* (communicating actual environmental input to the subject by the D-system). This is obvious. Indeed, integrative computation by the BAC firing mechanism works mostly when the real presynaptic input via the D-system to the neuron’s basal compartment is present (Larkum, 2013). Only when the cell is caused to fire by feedforward presynaptic D-input to the soma it becomes highly sensitive to the apical-dendritic modulation (Larkum, 2013). This means that the mechanism that brings contents to consciousness has its strong effect insofar as the actual input is present, meaning that in consciousness we clearly experience actual sensory input. Conversely, when subjects experience dreams or are engaged in daydreaming, the conscious contents that are hypothetically ignited by the presynaptic input to the dendrite tufts originating from more rostral, higher level cortical areas responsible for top-down effects (ignited by the L-system) cannot be as detailed and vivid as they are in the actual conscious perception of the real environment.

In the absence of top-down facilitation or selection by the L-system (which may be contolled by the brainstem-nonspecific thamocortical axis) the conscious contents are blurred or nonexistent. The necessary integration is missing. It can be presumed, that with increasing depth of anesthesia, from shallow sedation to deeper levels, the L-system and the D-system neural processes are changed in parallel: reduced state means reduced level of data input to the integrative system and vice versa. Even though strong inputs by the D-system may break through in the state of suboptimal L-system activity, they produce strong and focused sensory activation but in a limited, modality-specific local area (e.g., Laureys et al., 2002; Liu et al., 2013). However, integration with context from higher, supramodal associative processing centers, that are now silent, does not take place. Moreover, there are specific conditions, for example under the influence of hallucinogens, when the normal interaction of the L and D systems can be clearly dissociated.

Many subjective psychophysical phenomena of perception have temporal resolution with time constants varying around 30–300 ms (see Bachmann et al., 2011, for listing and characterizing the many pertinent phenomena.) Thus, for example metacontrast masking maxima occur typically at the target/mask delays of 30–60 ms; apparent motion in the form of beta motion integrating two static inputs for perception is optimized with SOA set at about 40–100 ms; iconic (visual sensory) memory (i.e., visible persistence) spans about 100–200 ms; critical flicker fusion temporal values are also specified in the range of dozens of ms; attentional blink is maximized at about 250–350 ms; target stimulus that is backward-masked by a pattern mask or by an other, competing, subsequently presented target becomes fully consciously available when the mask-free time approaches about 150 ms, but is replaced by mask in visual awareness with shorter target/mask time intervals. This suggests that the neural processes fitting to allow effective inter-stimuli interactivity (caused by presynaptic input arriving from different sources in response to briefly inset stimuli) and also for sustained activity enabling inter-stimuli interaction should be unfolding in the range of intervals of about 30–350 ms. In the traditional integrate-and-fire neuronal models, the EPSP decay is usually too short for this. However, in the BAC firing model the plateau-wave time-course corresponds well to this temporal scale. Most significantly, the BAC firing model nicely corroborates the distinction between the L-system effects and D-system effects related, respectively, to the presynaptic input to the tuft dendrite compartment and the basal somatic compartment of the post-synaptic membrane of layer-5 pyramids. Several psychophysical phenomena can be explained by the hypothetical action of the BAC firing mechanism allowing to combine the L-system and D-system effects by a single cellular level mechanism of integration. We believe that this is exactly the locus where the two traditions of NCC research can be mutually harmonized.

In the *visual backward masking by pattern*, a brief target stimulus (e.g., with 40 ms duration) is immediately followed by a masking pattern and as a result, target is not consciously perceived while the masking pattern is explicitly experienced (Bachmann, 1994; Breitmeyer and Öğmen, 2006). Recall that the theoretically necessary condition for sensory input to be integrated up to the level of conscious experience requires that input from feedforward D-channels targeted at the somatic compartment of the neurons that represent the stimulus content and associative (modulating) input targeted at the distal dendritic compartment that modulate content-specific activity up to the necessary level for consciousness must simultaneously coincide in time. Target signals feed synaptic receiving membrane compartment close to cell soma with a short delay (say, 30–50 ms) generating few somatic Na^+^ spikes. However, because this delay is too short for any associative input to arrive to the tuft region of the dendrite in response to the target-evoked perturbation, initially there is no target experience. Processing is pre-conscious. After some more time has lapsed, this associative, tuft-area directed presynaptic input arrives (say, with about 100 ms post-target delay), but it coincides with the mask-stimulus evoked Na^+^ spikes produced by the neurons that encode mask features. Because certain features of target and mask are shared by the target-responsive and mask-responsive cells (e.g., spatial location, some blob- or line-defining features, etc.), mask-responsive cells receive also the associative presynaptic Ca^2+^activity initiating input to the dendrite’s upper compartment (this was evoked by the preceding target) and as this input is coincident with Na^+^ spikes, a plateau wave is produced primarily for the neurons representing the mask features instead of the neurons representing exclusively the target features. We must remember here that the modulating input through the L-system in response to a perturbation by a stimulus has a longer delay to reach the apical compartments of the dendrites of the neurons compared to the delay it takes for the initial basal input throught the D-system to arrive to the cell. This basic fact is the crucial precondition for BAC firing based binding of the later presented stimulus with conscious representation instead of the first presented, briefly offset stimulus. Thus, the target-evoked dendritic Ca^2+^ mediated EPSP appears after a delay, is spread also to the dendrites of other cells (e.g., mask-related neurons), and coincides with the fast Na^+^ based somatic EPSP/spiking process of the mask-related cells. It is exatly then and there when and where the coincidence detection device sets in, but as a result, masking stimulus is emphasized for awareness. Bachmann (1994, 1997) in his model of modulated EPSPs as the explanatory mechanism of masking contends that temporal dynamics of the target-evoked N1 may be a signature of the L-system effects as they unfold in real time and are applied onto the D-system neural nodes.

In the *mutual masking of the spatially overlapping two successive targets* subjects are asked to report both successive stimuli, S1 and S2 (Bachmann and Allik, 1976; Bachmann, 1994). With very short SOAs (e.g., 0–20 ms) the results typically show neither the perfect identification of both the S1 and S2 nor the perfect masking of S1 and S2. The BAC firing mechanism based explanation is as follows. Because the SOA is so short, the L-system presynaptic input to the dendrites tuft-region compartment arrives with a considerable delay and “finds” S1 and S2 related neurons producing some residual somatically ignited Na^+^ spikes in response to the presynaptic D-system afferents. As a result, a plateau wave with enhanced spiking in the form of a burst is produced and both stimuli become bound together as an integrated pseudo-object in visual awareness. With intermediate SOAs (e.g., 40–90 ms) usually the following stimulus (S2) is well perceived, but the preceding stimulus (S1) is not perceived or not so well perceived. BAC firing mechanism based explanation of this psychophysical result is essentially the same as was used for explaining pattern masking above. When the time interval between onsets of S1 and S2 (SOA) exceeds about 100–150 ms, S1 becomes well visible also. The strongest dominance of S2 over S1 is typically found with SOAs around 40–60 ms. The BAC-based scenario for explaining this is as follows. Somatic presynaptic inputs from S1 carrying the contents of S1 arrive at the layer-5 neurons that represent S1 features, creating Na^+^ EPSPs’spiking response, but because there is no dendritic calcium response as yet (this response cannot be produced unless more time-consuming L-system mediated signaling originating from the nonspecific reticulo-thalamic influence on apical dendrites is present), plateau/burst wave is not produced for the neurons carrying S1 sensory contents in this early time window. When this delayed presynaptic input arrives at the distal parts of the apical dendrites, EPSPs of the S1-neurons are somewhat decayed, firing frequency is decreased, and probability of the conspicuos plateau-wave is low. S1 is not explicitly perceived so well as the following S2 which altogether suppresses S1 in conscious perception. This is because the somatic-compartment response of the following stimulus related neuron (S2 neuron) is maximized at the moment temporally coinciding with the slower (S1-induced) calcium wave and because due to the spatial-location and some partial featural similarity between S1 and S2, apical dendrites of the S2 neurons are also targeted and the plateau wave is distinctive and strong for the S2-related neurons. As a result, S2 is consciously perceived and S1 perception suppressed. (It is likely that some additional assumptions with regard to the mechanisms involved are needed. For example, the role of NMDA receptors and finely localized sub-cellular membrane mechanisms of competitive inhibition could be postulated to explain why S2 so robustly overpowers S1. See Larkum et al. (2009), for premises of this kind of mechanisms. Also, more precise understanding of the workings of GABAa and GABAb mediated inhibitory mechanisms may be needed in order to precisely describe the ways the L-system and D-system interact on the basis of the layer-5 pyramidal neurons.)

*Metacontrast masking* can be explained similarly by the L-system and D-system interaction taking place at the basal and apical-dendritic membrane of the cortical neurons representing the contents of stimulation. In this phenomenon, S1 (the target stimulus) is spatially non-overlapping with S2 (the masking stimulus), but is closely spatially adjacent. Typically, when contrasts and durations of S1 and S2 are compatible, with very short SOAs S1 and S2 are both perceived as if they form a single, composite object. The BAC firing based L/D interaction explanation is similar to what was used for mutual masking with shortest SOAs as described above. Simply because in metacontrast S1 and S2 are not spatially superimposed, the common formed perceptual image allows good discrimination of mask and target features spatially. It is easy to envisage a small neural network where the distinct spatial location cues dictate that modulations executed by the associative presynaptic input to the apical dendritic compartment is bifurcated to different neurons in parallel due to the difference in the receptive fields which is an important feature of stimulation. With intermediate SOAs (e.g., 40–70 ms) S1 is again strongly masked while S2 is well perceived – metacontrast masking is effective. The theoretical explanation is similar to the one presented when explaining pattern- and mutual masking. Moreover, as the shapes of S1 perimeter and S2 internal part surrounding S1 shape are identical or very similar in most of the metacontrast stimuli setups, surface quality of S2 (i.e., the metacontrast mask such as a ring for example) becomes preceptually represented combined with S1 shape which lacks its surface quality and therefore is shown as being “emptied” from this quale.

Explaining masking based on L-system and D-system interaction is not a recent “invention.” For example, Bachmann (1984, 1997) in his perceptual retouch theory interpreted masking as a result of L-system-mediated modulation of mask-specific signals up to the level of conscious experience by simultaneously depriving the target-specific signals of a sufficient level of this kind of modulation. Because L-system effecs at the cortical level of the D-system nodes are delayed compared to the initial D-system effects, the boost of L-system modulation ignited by target presentation is maximized exactly when the freshly ariving D-system signals carrying mask content arrive cortical neurons dedicated to encode contents. As the signal-to-noise ratio of the mask-related specific afferent activity is higher than the specific target-related activity when the boost of L-modulation arrives, mask-related net activity combining the presynaptic D-input and L-input effects wins over the target-related net activity (target-related neural activity has decayed already when L-input arrives, but mask-related activity is maximized at that moment). Modulated post-synaptic EPSPs (Bachmann, 1994, 1997) and N1 components of ERPs (Bachmann, 1984) were used to illustrate the hypothetical D-system and L-system interactive effects producing psychophysical effects of visual awareness. Results of more recent studies with directly measuring ERPs and spectral EEG perturbations and using contrastive analysis with invariant physical stimulation conditions also show that NCC in masking includes a relatively late brain activity (Aru and Bachmann, 2009a,b).

A psychophysical paradigm called *perceptual latency priming* (PLP) has helped to demonstrate that there is a relative latency advantage (i.e., earlier perception) of a visual stimulus that is preceded by another, masked stimulus at its location (Bachmann, 1989; Neumann and Scharlau, 2007; Scharlau, 2007). The first stimulus accelerates perception of the second stimulus even if the first stimulus is backward-masked by the second one up to a total invisibility for direct awareness. In the control condition S2 is presented alone and the temporal delay of its perception is measured psychophysically (e.g., temporal order judgment against a reference stimulus). In the main condition, S2 is preceded by a priming stimulus S1 (spatially adjacent or overlapping with S2). As a result of priming, subjective delay is shortened compared to the control condition without priming. (The effect is obtained also when the prime remains unconscious and is masked.) L- and D-systems interaction-based explanation capitalizing on BAC firing neuronal mechanism assumes that S1 induces the processes consisting in presynaptic somatic input to the neurons encoding its features and a temporally delayed associative (collateral and/or cortico-thalamic) presynaptic input to apical tufts of the neurons that represent attributes of the stimuli associated with the prime in an associative network (including S2 related neurons). Spatial location and approximate size are among the important attributes shared by the prime (S1) and the target (S2). This delayed apical presynaptic input sent via the L-system appears to be temporally coincident with S2 related early somatic-compartment activity in the neurons representing S2 (this being brought about via the D-system). As a result, a plateau wave with a burst of spiking will be an associative medium for temporal binding of S1-evoked late activity and S2-evoked early activity. Because the apical-compartment input for the S2-representing neurons is unusually early (due to the preceding S1-evoked associative activity), already the very first somatic presynaptic input to the S2 neurons finds unusually early coincidence with the apical calcium spikes, which means that S2 is consciously perceived unusually fast.

*Object substitution masking* (OSM) can be explained in a similar vein. In OSM, if a backward mask does not cover the target in space and is spatially and form-wise sparse (e.g., four dots surrounding a target image such as a landolt C), the masking effect is absent. However, when the same target and mask are presented among the spatially distributed distractor objects (with the subject not knowing beforehand where the target is located), with the mask specifying which object is the target and when mask offset is considerably delayed after target offset (a simultaneous onset, asynchronous offset display), strong masking occurs (Enns and Di Lollo, 1997; Di Lollo et al., 2000). In OSM, traditionally weak masks have strong effects when attention is not focused on the target before its presentation. The BAC firing mechanism could be used to suggest a similar explanation to what was presented for backward masking and mutual masking. However, it is necessary to explain why masking is absent in the condition without distractors and occurs when distractors are presented. It is natural to expect that the temporally delayed associative top-down modulation targeting presynaptically the layer-5 neurons’ dendrites and igniting the BAC firing mechanism is susceptible to the lateral inhibitory influences mediated by the inhibitory synaptic effects in the dendrites’ tuft compartment. If the early-onset OSM display contains many competing stimuli presented simultaneously from the several spatially separated locations, the subsequent delayed associative modulation of the target-related neurons will be inhibited by the lateral afferents from the formerly activated neurons representing the spatially remote competing stimuli. Basically, OSM results from the main share of the L-system-mediated modulation being used by the mask D-system representation instead of the target D-system representation.

In the *motion-induced blindness* displays a few static yellow disks otherwise clearly perceptible become extinguished from awareness from time to time, provided that they are viewed on the background of a moving texture or a set of moving random dots (Bonneh et al., 2001). The BAC firing mechanism-based explanation: two higher level representations – static disks versus moving set of small items – compete for being “serviced” by the top-down modulation by the L-system targeted at the tuft compartment of their respective neurons. Although somatic D-system responses to presynaptic input are preserved all the time for both types of stimuli, dendritic apical compartment afference through the L-system is unstable and fails at times. As a result, discs temporarily fade out of awareness. Competition at the higher level between two classes of neurons representing two different actual perceptual events (one showing static objects and the other a dynamic background) does not allow to send sufficiently steady and efficient top-down signals simultaneously to the dendrites of the lower level neurons that represent local cues of these two types of events.

*Ganzfeld effects*. When a subject stares at an empty field with homogeneous coloring (no brightness gradients and contours in it) for a long time, sooner or later an experience of “fading out” or “emptiness” of the visual sense occurs (Avant, 1965; Wackermann et al., 2008). Colorful visual experience disappears. The explanation founded on the L/D systems’ interaction implemented by the BAC firing mechanism assumes this: without almost any specific input that would “recruit” layer-5 pyramidal neurons by somatic-compartment presynaptic input (there is simply not enough sensory input) being available, apical-compartment directed modulation by the L-system becomes without its necessary somatic counterpart of presynaptic input and as a result, any sufficient amount of the plateau-waves is absent. Awareness “gets visually empty.” This interpretation invites another assumption. The top-down modulating signals from the higher level are generated insofar as there is a certain minumum amount of object- and feature-specific variability in the perceived environment. (Hypnotic states resulting from sensory input monotony and lack of variability are also a common reality. Conversely, hallucinatory experiences so common to Ganzfeld experiments suggest a dominance of the top-down or associative input mediated by the dendritic calcium sub-mechanism in predetermining the contents of consciousness in comparison with the input from the actual sensory environment.) How exactly this principle is implemented in the brain remains only a matter of speculation for now.

**FIGURE 1:**
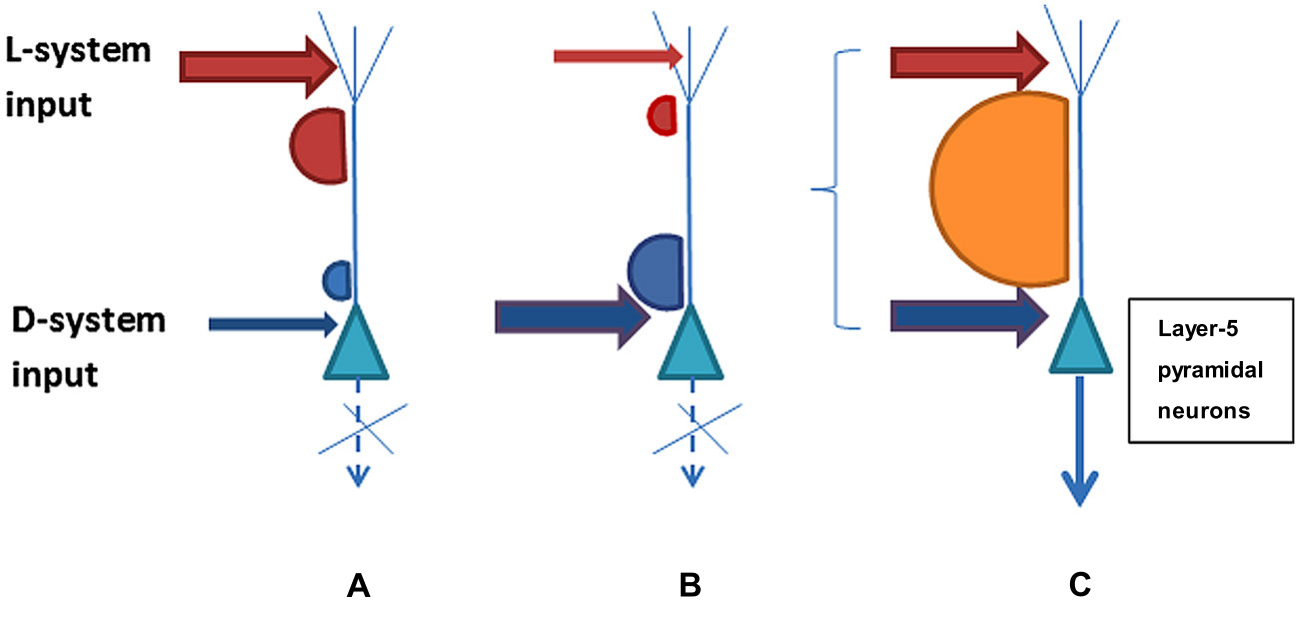
**Three possible variants of L- and D-system presynaptic input to layer-5 pyramidal neurons that may determine contentful conscious experience.** Variant **(A)** shows lack of sufficient acitivity for long-range integration and consciousness because D-system specific input to the perisomatic compartment of pyramidal neurons is weak or asynchronous with L-system presynaptic inputs to the neuron’s apical compartment. Variant **(B)** illustrates the opposite situation where the D-system presynaptic input is present but the L-system contribution is weak or incoincident. Both **(A)** and **(B)** represent scenarios lacking consciousness with its contents. In contrast, variant **(C)** illustrates sufficiently strong and synchronous presynaptic input from the D- and L-systems that leads to plateau-wave of activity and integration of the neuron’s contentful contribution to the phenomenal field of consciousness.

The possible combinations of the D-system and L-system neurons’ post-synaptic activity in response to the strength and synchronicity of their presynaptic inputs are schematically illustrated in **Figure 1**. The psychophysical phenomena of variable consciousness can be explained on the basis of the variants of D/L interaction. Variant (i) characterizes conditions for an absence of contentful conscious experience when the D-system input is insufficient or asynchronous with the L-system despite a strong contribution from the latter. Variant (ii) shows how conscious experience is absent when the L-system contribution is sub-par and/or asynchronous despite the normal contribution of the D-system. Variant (iii) illustrates the emergence of the interactivity between D- and L systems sufficient for conscious awareness as a result of strong enough and synchronized input.

## CONCLUSION

In this article, we presented a theoretical perspective, supported by variable empirical data, integrating the two traditions of NCC research which have been developing in relative mutual isolation. Tradition-1 has been examining consciousness state systems with their central role in regulating the level of consciousness, but largely ignoring or overlooking, or being unable to study in detail the functioning and expression of the system of specific contents. Tradition-2, on the other hand, has produced a vast amount of information about the system of processing contents of consciousness and its activity signatures, but has largely ignored the involvement of the state/level system. Importantly, there is enough scientific knowledge about the build and expression of function of the neural mechanisms subservig each of these two functions but unfortunately, there has been insufficient research simultaneously investigating the contributions of the mechanisms serving the two functions within the same experiment in the context of contrastive analysis. We suggest an integration of these two paradigmatic perspectives whereby the objective measurements of the processes of the two neural systems – the state/level system and the contents system – are carried out in experiments where the confounds from stimulus variability are avoided and the problems emerging from the need to distinguish NCCpr, NCC, and NCCae are minimized.

We describe the two neural systems – the data-representng D-system and the level-modulating L-system – and characterize their working parameters and regularities. We suggest a heuristic “formula” C = L × D enabling to combine the two necessary subsystems for consciousness jointly sufficient for producing the subjectve experience with its specific content and varyig in the level of expression of this content, spanning from unconscious to fully conscious and stable phenomenal representation. Importantly, several measurable neurobiological, temporal, and spatial parameters of functioning of the two systems allow us to explain many experimental facts as a result of interaction of the L- and D-systems in real time. Our conceptualization is consistent with several well known approaches and accepted principles in consciousness studes such as the importance of top-down, backpropagating information flow in the brain for consciousness, the temporally delayed nature of phenomenal experience compared to unconscious specific information processing and the importance of the long-range connectivity and global integration of information for consciousness. Among other aspects of the present theoretical view, it must be emphasized that because the L-system-based modulation targets superficial cortical layers and thus largely contributes to negativity and because long-range connectivity which is principally significant for the information integration theory of Tononi and associates also implies apical presynaptic input, the SCP/negativity hypothesis, info-integration theory, and Larkum’s BAC firing mechanism seem highly mutually consistent. We also think that our approach has merit because the neural subcellular level, cellular-level, local field potential level, local-circuit level and global-connectivity level aspects seem to be harmonized within the same conceptual framework, which at the same time, does not necessarily remain merely speculative but can be tested and falsified by specific experiments combining brain imaging and psychophysical experiments. We believe that C = L × D helps better specify the NCC, especially by objectively measuring and differentiating the relative contribution of the mechanistically distinguishable subcomponents of the brain involved in producing the astonishingly rich and often hearthbreakingly beautiful phenomenal view of the world.

## Conflict of Interest Statement

The authors declare that the research was conducted in the absence of any commercial or financial relationships that could be construed as a potential conflict of interest.
